# Enabling Delay of Gratification Behavior in Those Not So Predisposed: The Moderating Role of Social Support

**DOI:** 10.3389/fpsyg.2016.00366

**Published:** 2016-03-18

**Authors:** Xiaoyan Liu, Lei Wang, Jiangqun Liao

**Affiliations:** ^1^College of Business Administration, Guangzhou UniversityGuangzhou, China; ^2^Department of Psychology, Peking UniversityBeijing, China; ^3^Department of Psychology, Tsinghua UniversityBeijing, China

**Keywords:** delay of gratification, social support, expression of DG Behavior, moderator

## Abstract

The presence of delay of gratification (DG) in childhood is correlated with success later in a person's life. Is there any way of helping adults with a low level of DG to obtain similar success? The present research examines how social support helps those low in DG nonetheless to act similarly to those high in DG. This research includes both correlational studies and experiments that manipulate social support as well as both field studies and a laboratory study. The results show that with high social support, employees (Study 1) and university students (Study 2) low in DG report vocational and academic DG behavioral intentions, respectively, similar to those high in DG. Study 3 found that participants low in DG who were primed with high social support expressed job-choice DG similar to those high in the DG. Study 4 controlled for mood and self-image and found that participants low in DG who were primed with high social support expressed more money-choice DG than those high in the DG. Study 5 showed that social support moderated the relationship between DG and actual DG behaviors. These findings provide evidence for a moderating role of social support in the expression of DG behavior.

## Introduction

It is now a well-known finding that a 4 year old child who resists the temptation to eat a marshmallow immediately in order to get two marshmallows 15 min later experiences later-life success in such diverse areas as SAT scores, health, and marriage (see Ayduk, 2007; for review, see Schlam et al., 2013). But what about the children who could not resist the marshmallow? Is there any way of helping adults with a generally low level of delay of gratification (DG) to obtain similar success in life?

Mischel (1974) defined DG as a choice-orientation in which individuals willingly abandon instant gratification for more valuable long-term goals. DG is considered an integral part of self-regulation in one's personality and a sign of maturation, Adolescents and adults low in the DG trait show less stress tolerance, social competence, and planning competence (Mischel et al., 1988; Shoda et al., 1990). These findings raise an important question: are there any external resources that can help people low in the DG trait to compensate for such disadvantages? Social support may be one such resource. As an external and interpersonal coping resource (Seeman, 1996), social support can increase ability to overcome frustration and difficulty (Sarason et al., 1983). We propose that for individuals low in the DG trait, social support may lead to DG behavioral intentions and behaviors like those seen in people who are high in the DG trait.

Existing measures of adolescent and adult DG actually measure their behavioral intention to delay gratification rather than a general DG disposition (Funder and Block, 1989; White et al., 1994; Wulfert et al., 2002). That is, the participant is usually given a series of hypothetical choices, such as receiving $5 now vs. $10 one week later. In contrast, we measure a general DG trait as well as DG behavior in specific realms. We then explore the moderating effect of social support on the expression of DG behavior.

## Influences on the expression of DG behavior

The development of the DG trait during childhood appears important for a person's entire life. Research indicates that 4-year-old children who are more able to delay gratification are able to achieve higher academic scores (Mischel et al., 1992; Duckworth and Seligman, 2005) and cognitive control (Eigsti et al., 2006) as adults. As adolescents they exhibit more concentration and frustration tolerance than their peers (Mischel et al., 1988; Shoda et al., 1990), and they are perceived as more interpersonally competent by parents and peers (Mischel et al., 1989). In addition, DG has been found to be a fundamental protective mechanism that shields highly rejection–sensitive individuals from negative interpersonal difficulties like aggression and peer rejection (Ayduk et al., 2000). Adolescent and adults' behavioral intentions of DG are also important for their work, learning, and everyday lives. High school students who choose delayed payment in order to receive more money show less involvement with cigarettes, alcohol, and marijuana than those who choose immediately available payment with less money (Wulfert et al., 2002). University students with high academic DG show motivation and learning strategies more often than others (Bembenutty and Karabenick, 1998, 2004; Bembenutty, 1999), and academic DG was a mechanism in the relationship between students' self-efficacy and their academic achievement (Bembenutty, 2002). DG appears to be an important dimension of the work ethic, and people high in DG show better performance (Furnham, 1987; Miller et al., 2002). In addition, vocational DG helps explain how career management relates to job satisfaction (Liu et al., 2007).

What influences DG behaviors? Although some physiological control is related to DG (such as refraining from blinking and tolerating a painful stimulus; Hoyle, 2006), most DG behaviors are neither so simple nor so direct as these physiological indices. Both situational and intrapersonal factors may affect the expression of DG. For example, DG behaviors tend to be accompanied by time discounting. As waiting time increases, people become more reluctant to choose delayed outcomes (Hesketh et al., 1998; Frederick et al., 2002); however, if waiting time is filled with casual reasoning, the discounting value of the delayed outcome decreases (Hesketh et al., 1998). When people know that others will supervise or pay for their behavioral outcomes, they exhibit more self-control behaviors in order to obtain long-term benefits; even when less motivated to overcome difficulties, they consistently tend to choose to pursue long-term goals under external control situations (Fishbach and Trope, 2005).

Adults (44 years and older) at the stabilization and maintenance career stages who were viewed as cautious and long-term orientated were more reluctant to delay gratification in their careers than younger people (less than 31 years old). Perhaps during their longer experience, they found delayed gratification did not always lead to more rewards, or perhaps they felt they had less time for delay (Pogson et al., 2003). Individuals who believe that the world is a just place where people get what they deserve have less desire for smaller, immediate rewards at the expense of larger, delayed rewards (Callan et al., 2009). These findings all reflect that DG behavior is a complex process that cannot simply be predicted by a monolithic DG trait.

## The moderating role of social support in the expression of DG

Social support refers to “perceived or actual instrumental and/or expressive provisions supplied by the community, social networks, and confiding partners” (Lin, 1986), and has long been regarded as a coping resource, a kind of buffer between stressful life events and the development of symptoms (Seeman, 1996). For instance, in times of crisis, the social support from a religious community can help individuals overcome adversity and social rejection (Haden et al., 2007; Aydin et al., 2010), and social support from spouses can bring positive outcomes for patients with chronic illness (Franks et al., 2006; Iida et al., 2010). Recent research shows that receiving social support from fellow group members leads employees to make a greater effort in group work and performance (Wessolowski et al., 2014). Thus, notably, social support can improve the capability to overcome frustration and difficulty (Sarason et al., 1983).

Delay of gratification is a kind of self-regulation that often needs planning, controlling, and waiting, According to the theory of Regulatory Depletion Patterns, an individual's acts of volition (e.g., choice, active response, and self-regulation) draw on limited resources. For example, people who resist the impulse to eat tempting chocolates but eat radishes instead subsequently give up much faster on a difficult, frustrating puzzle task than did do people who had been able to indulge the same impulse to eat chocolate (Baumeister et al., 1998). People need some form of energy or other ways to extend their personal resources in order to accomplish such acts of volition (Muraven et al., 1998). Moreover, people with fewer resources should therefore be more likely to conserve the resources they have compared with those with greater resources (Muraven et al., 2006). In this case, we consider DG to be such a resource; those low in DG are more likely not to exercise DG behavior than those higher in the trait. Social support, however, may be able to help buffer or moderate the impact of mental depletion and provide the extra energy needed for DG behavior. We thus put forward that under a high social support condition, individuals low in DG would nonetheless show relatively high DG behavioral intentions or behavior—as much as those high in DG; while under low social support conditions, individuals low in DG would be less likely to defer gratification than those with a high level of DG.

## Present research

We used several different methods and dependent variables to evaluate the role of social support in the expression of DG behavior and to demonstrate the generalizability of our findings. In Study 1, we tested the moderating role of social support between general DG t and vocational DG behavioral intention among employees. In Study 2, we tested the moderating role of social support between general DG and academic DG behavioral intention among university students. The next three studies were experimental. Study 3 manipulated social support and established the moderating role of social support between general DG and job-selection DG. In Study 4, we additionally controlled for mood and self-image, and verified the moderating role of experimentally manipulated social support between general DG and monetary choice DG. Study 5, a laboratory investigation, measured DG behaviorally and again showed that social support moderated between general DG and actualDG behaviors. The five studies were carried out in accordance with the recommendations of the Ethics Committee of Peking University, with written informed consent from all subjects. All subjects gave written informed consent in accordance with the Declaration of Helsinki.

## Study 1

Study 1 used self-report questionnaires to initially explore the hypothesis whether social support moderates the relationship between the DG trait and DG behavioral intention. Since most researchers have found that perceived social support is a better predictor of psychological status than objectively measured social support (Barrera et al., 1981; Sarason et al., 1985) and that social support was beneficial only when the support was responsive (Maisel and Gable, 2009), we adopted a perceived social support measure for the questionnaire studies.

### Methods

#### Participants

We recruited 354 employees (50% male) from various industries with a mean age of 29.4 years (*SD* = 5.21, range from 18 to 62 years); 76.9% had a college diploma or higher degree. Their mean length of service was 3.3 years (*SD* = 2.81, range from 1 to 27 years).

#### Procedure and materials

We adopted a snowball sampling technique in which we sent the survey out to contacts who worked in various companies, including state-owned and foreign enterprises in China. This recruitment e-mail contained study information, the link to the survey, and instructions to forward the e-mail to friends and colleagues who worked in similar companies. Using this approach, the 100 initial e-mails yielded 354 responses within 2 weeks. The respondents voluntarily filled out the questionnaires online.

The online questionnaire consisted of measures for the DG trait, social support, and DG behavioral intention.

##### General delay of gratification

The 12-item General Deferment of Gratification Questionnaire (*GDGQ*, Ray and Najman, 1986) was used, with items (e.g., “I enjoy a thing all the more because I have to wait for it or plan for it”; α = 0.76) rated on a 7-point Likert scale (1 = *very strongly disagree*, 7 = *very strongly agree*).

##### Perceived social support

The 12-item Multidimensional Scale of Perceived Social Support *(MSPSS;* Zimet et al., 1988) can be scored to measure perceived support from family, friends, and a significant other, or global perceived support. We used the overall scale to measure total perceived social support. Items (e.g., “I can count on my friends when things go wrong”; α = 0.91) were rated on a 7-point Likert scale (1 = *very strongly disagree*, 7 = *very strongly agree*).

##### Vocational DG behavior (VDG, Liu et al., 2007)

VDG is a vocation choice orientation according to which instant gratification such as rest, recreation and other activities that are not beneficial for present work is willingly abandoned for a series of more long-established goals such as better accomplishing working tasks, and achieving more rewards and much higher standards (Liu et al., 2007). The 8-item VDG Questionnaire was used, with items (e.g., “It isn't a problem to start my career as an ordinary clerk as long as there is a promotion possibility”; α = 0.73) rated on a 7-point Likert scale (1 = *very strongly disagree*, 7 = *very strongly agree*).

### Results and discussion

**Figure 1:**
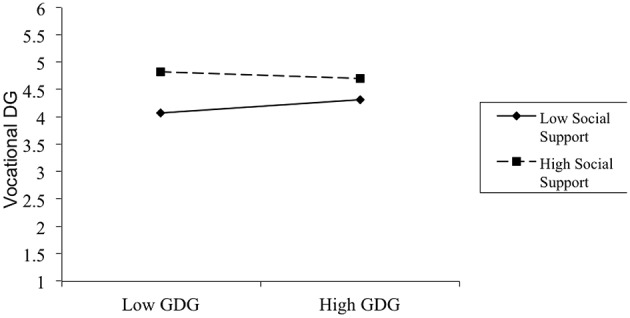
**Interaction between GDG and PSS on vocational DG**. Note: low = 1 SD below the mean; high = 1 SD above the mean.

See Table 1 for means, standard deviations, and correlations for all variables in Study 1. We tested the hypothesis with hierarchical regression analyses (see Table 2). Both the GDG and PSS were centered, and the interaction term was based on the product of these centered variables (Cohen et al., 2003). In Step 1, VDG was regressed on the control variables including gender, age, education, length of service, and position. We found that these control variables accounted for 4.0% of the variance in VDG, *F*_(5, 347)_ = 2.84, *p* < 0.05, and gender predicted VDG such that males had higher VDG than females. In Step 2, VDG was regressed on the PSS and GDG. This step produced significant results, *R*^2^ = 0.14; ▵ *R*^2^ = 0.10, *F*_(7, 345)_ = 8.11, *p* < 0.01, which showed that PSS was a significant predictor of VDG such that as PSS increased, so did VDG β = 0.32, *p* < 0.01). But GDG was not a predictor for VDG. In Step 3, VDG was regressed on the two-way interaction between GDG and the PSS. The interaction term was a significant predictor of VDG, *R*^2^ = 0.15; ▵*R*^2^ = 0.01, *F*_(8, 344)_ = 7.74, *p* < 0.01 (see Figure 1).

**Table 1:** **Means, standard deviations, and correlations for Study 1–5**.

**Study 1**	***M***	***SD***	**1**	**2**	**3**	**4**	**5**	**6**	**7**	**8**
1 Gender	1.50	0.50	−							
2 Age	29.38	5.21	−0.19^**^	−						
3 Education	2.95	0.75	−0.09	0.23^**^	−					
4 Length of service	3.25	2.81	−0.08	0.41^**^	−0.05	−				
5 Position	1.69	0.93	−0.12^*^	0.40^**^	0.14^**^	0.16^**^	−			
6 GDG	4.68	0.77	−0.17^**^	0.23^**^	0.11^*^	0.12^*^	0.15^**^	−		
7 PSS	5.20	0.97	0.12^*^	0.00	0.07	−0.10	−0.05	0.17^**^	−	
8 VDG	4.76	0.91	−0.12^*^	−0.12^*^	−0.04	−0.09	−0.09	0.07	0.30^**^	−
**Study 2**			**1**	**2**	**3**	**4**	**5**			
1 Gender	1.61	0.49	−							
2 Age	20.57	0.54	0.12	−						
3 GDG	4.71	0.82	0.04	−0.05	−					
4 PSS	5.05	1.07	0.13	−0.03	0.26^**^	−				
5 ADG	2.83	0.49	0.03	−0.06	0.38^**^	0.27^**^	−			
**Study 3**			**1**	**2**	**3**	**4**	**5**			
1 Gender	1.50	0.50	−							
2 Age	21.74	0.73	−0.01	−						
3 GDG	4.92	0.74	−0.16	0.05	−					
4 Social support	0.55	0.50	0.07	−0.20	−0.09	−				
5 Job-choice DG	5.62	1.62	0.11	−0.24	0.29^**^	0.09	−			
**Study 4**			**1**	**2**	**3**	**4**	**5**	**6**	**7**	
1 Gender	1.53	0.50	−							
2 Age	21.03	0.89	−0.07	−						
3 Positive affect	3.30	0.59	0.01	0.02	−					
4 Negative affect	3.14	0.83	−0.10	0.10	0.27^*^	−				
5 Self-image	4.19	0.72	0.07	−0.19	−0.01	−0.48^**^	−			
6 GDG	4.83	0.70	0.12	0.33^**^	0.05	0.02	0.12	−		
7 Social support	0.53	0.50	−0.00	−0.07	0.11	0.18	−0.27^**^	−0.31^*^	−	
8 Money-choice DG	3.83	1.77	0.05	0.27^*^	0.10	0.04	0.12	−0.05	0.07	−
**Study 5**			**1**	**2**	**3**	**4**	**5**	**6**	**7**	
1 Gender	1.63	0.49	−							
2 Age	22.13	2.81	−0.20	−						
3 Income	3.84	1.05	0.20	−0.14	−					
4 Positive affect	2.96	0.74	−0.09	−0.16	−0.09	−				
5 Negative affect	1.94	0.62	−0.04	0.18	−0.10	−0.33	−			
6 GDG	4.69	1.09	−0.32	0.02	−0.37^*^	0.63^**^	−0.36^*^	−		
7 DG task	0.47	0.51	−0.05	0.27	−0.28	−0.03	0.23	0.08	−	
8 Social support	0.24	0.05	0.24	0.05	0.27	−0.29	−0.12	−0.05	−0.13	−

Note. Gender: males = 1; females = 2; Education: senior high school education = 1, junior college school education = 2, undergraduate education = 3, master education = 4. Position: employees = 1, front-line manager = 2, mid-level manager = 3, senior manager = 4.GDG = general DG; PSS, perceived social support; ADG, academic DG; VDG, vocational DG; Social Support (Studies3–5): 1 = high social support, 0 = low social support. DG task; 0 = getting ¥5, 1 = getting ¥15.

* p < 0.05;

** p < 0.01.

**Table 2:** **Regression analyses for Studies 1–4**.

	**Variable**	**Study 1**		**Study 2**		**Study 3**		**Study 4**	
		**β**	***t***	**β**	***t***	**β**	***t***	**β**	***t***
First step	Gender	−0.11	−2.76^**^	−0.03	0.45	0.1	0.8	0.11	0.5
	Age	−0.15	−1.65	−0.07	−0.87	−0.24	−1.87	0.50	2.21^*^
	Education	−0.02	−0.39						
	Length of Service	−0.04	−0.73						
	Position	−0.05	−0.93						
	PA							0.15	0.66
	NA							0.11	0.37
	Self-image							0.20	0.74
	Δ*R*^2^		0.04		0.01		0.07		0.09
	*F*		2.84^*^		0.44		2.11		
Second step	GDG	0.03	0.48	0.16	4.66^**^	0.57	2.79^**^	0.57	2.79^**^
	Social support	0.32	6.15^**^	0.09	2.67^*^	0.06	0.47	0.18	1.46
	Δ*R*^2^		0.10		0.17		0.12		0.04
	*F*		8.11^**^		9.68^**^		3.16^*^		1.22
Third step	GDG × Social support	−0.11	−2.14^**^	0.15	−2.12^**^	0.33	−2.14^**^	−0.61	−2.02^*^
	Δ*R*^2^		0.01		0.02		0.06		0.06
	*F*		7.74^**^		8.79^**^		3.62^**^		1.64^*^

Note: Study 1 dependent variable, Vocational Delay of Gratification; Study 2 dependent variable, Academic Delay of Gratification; Study 3 dependent variable, Job-choice Delay of Gratification; Study 4 dependent variable, Money-choice Delay of Gratification.

* p < 0.05;

** p < 0.01.

To examine the nature of these interactions, we conducted simple slopes analyses as recommended by Aiken and West (1991). The simple slopes of the two lines (high vs. low PSS; low = 1 SD below the mean; high = 1 SD above the mean) were then calculated and tested for statistical significance (i.e., whether the slopes were significantly different from zero). Among participants who scored low in PSS, GDG was a relatively significant predictor of VDG (β = 0.13, *p* = 0.05), but among those who scored high in PSS, GDG was not a significant predictor of VDG (β = −0.06, *p* > 0.10).

In summary, the first study provided initial evidence that relationship between DG trait and VDG behavioral was moderated by social support. In the high social support condition, employees with a low level of the DG trait displayed as much vocational DG behavioral intention as those high in the DG trait, but in the low social support condition, employees high in GDG displayed more VDG than those in low GDG. This effect persisted when gender was statistically controlled for. To examine the generalizability of the findings, a second study was conducted to test whether social support could provide the resource for individuals low in the DG trait to display DG behavior in the academic domain.

## Study 2

The objective of Study 2 was to examine the generalizability of the findings from Study 1 by testing an independent sample of university students with a new dependent variable: academic delay of gratification (ADG). This kind of DG refers to “students' postponement of immediately available opportunities to satisfy impulses in favor of pursuing chosen important academic rewards or goals that are temporally remote but ostensibly more valuable” (Bembenutty and Karabenick, 1998). In Study 2, we adopted an ADG scale to measure the DG behavioral intentions of university students. We assumed that under low social support conditions, students high in the DG trait would display more ADG than those low in the DG trait, while under high social support conditions, students low in the DG trait would exhibit as much DG behavior as those high in the DG trait.

### Methods

#### Participants

One hundred and eighty-three students (39% male) from a large public university in China volunteered to participate in the study, with ages ranging from 19 to 22 years old (*M* = 20.6, *SD* = 0.54).

#### Procedure and materials

Participants were recruited from introductory level psychology classes and were given a questionnaire packet ostensibly designed to assess various aspects of personality. Administration of the instruments took place in their traditional classrooms. The GDGQ (α = 0.73) and MSPSS (α = 0.91)that were used in Study 1 were also adapted to respectively measure the DG trait and perceived social support in Study 2.

##### Academic DG behavior (Bembenutty and Karabenick, 1998)

The 10-item academic delay of gratification scale was used, with each item using a two-option scenario. Option A offered more immediate gratification, such as “Going to a favorite concert, play, or sporting event, even though it may mean getting a lower grade on an exam in this class to be taken the next day,” and Option B that offered a delayed gratification, such as “Staying home and studying to increase your chances of getting a higher grade.” Students rated their preference of each option and responded on a 4-point scale: 1 = *Definitely choose A*, 2 = *Probably choose A*, 3 = *Probably choose B*, and 4 = *Definitely choose B* (α = 0.67).

### Results and discussion

**Figure 2:**
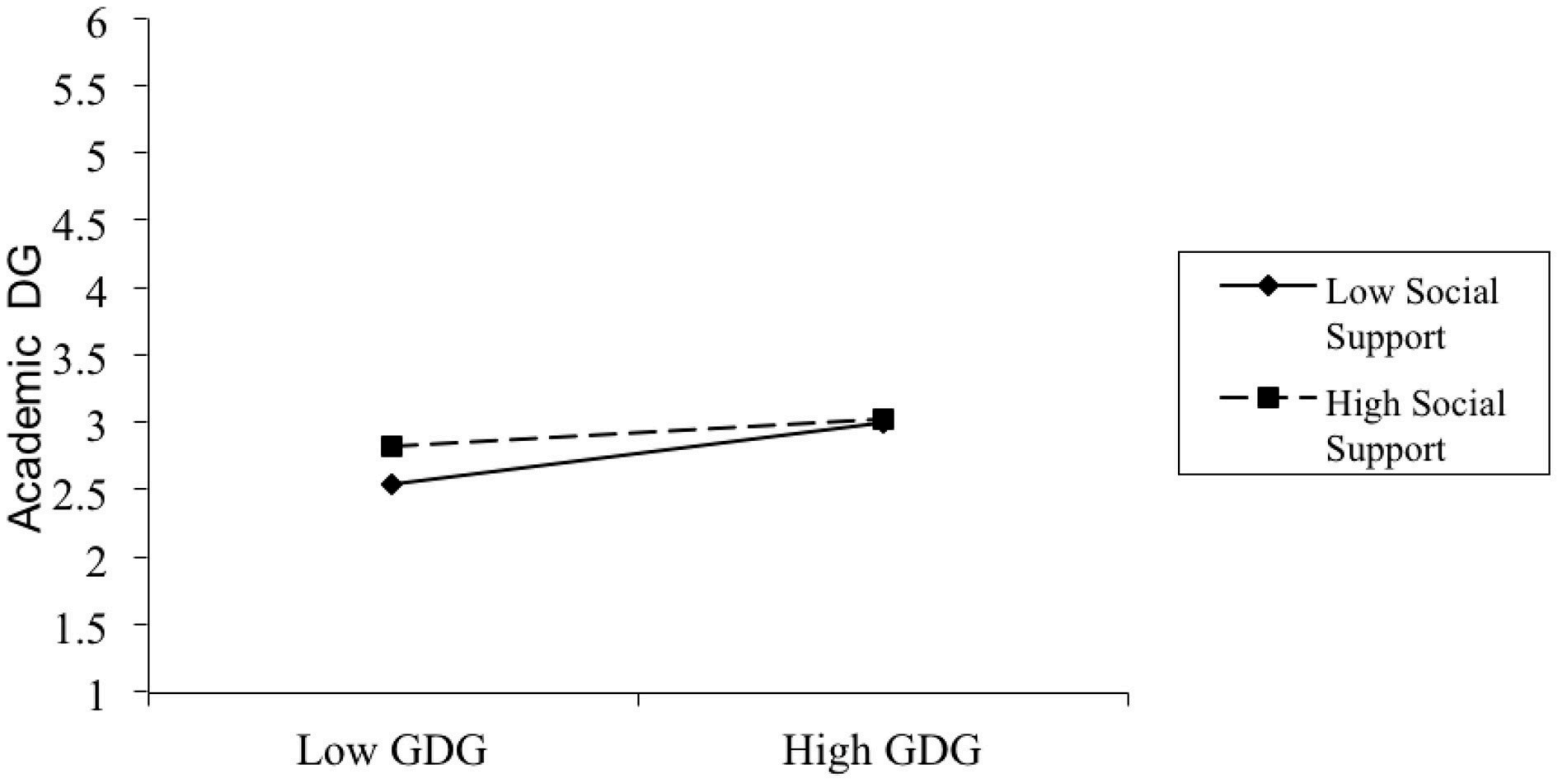
**Interaction between GDG and PSS on academic DG**. Note: low = 1 SD below the mean; high = 1 SD above the mean.

See Table 1 for means, standard deviations, and correlations for all variables in Study 2. GDG was positively related to ADG. In correspondence with Study 1, the hypothesis was also tested by means of hierarchical regression analyses. In Step 1, ADG was regressed on control variables including gender and age. The results from Step 1 did not reach significance, *R*^2^=0.01. In Step 2, ADG was regressed on the PSS and GDG. This step produced significant results, *R*^2^ = 0.18; ▵*R*^2^=0.17*, F*_(4, 178)_ = 9.68, *p* < 0.01, which showed that both GDG and PSS were significant predictors of ADG (β = 0.16, *p* < 0.01; and β = 0.09, *p* < 0.01, respectively). In Step 3, ADG was regressed on the two-way interaction between GDG and the PSS, and the interaction term was found to be a significant predictor of ADG, *R*^2^ = 0.20; ▵*R*^2^=0.02, *F*_(5, 177)_=8.79, *p* < 0.01 (see Figure 2). Simple slopes analyses showed that for participants who scored high in PSS, the relationship between GDG and ADG (β = 0.10, *p* < 0.05) was weaker than for participants who scored low in PSS (β = 0.23, *p* < 0.01).

Taken together, Study 2 replicated the effects found in Study 1 with a distinct measure of DG behavioral intention. Once again, the results demonstrated that GDG was different from ADG, and social support moderated the relationship between the DG trait and academic DG behavioral intention. When perceiving enough social support, the university students low in the DG trait would show academic DG behavioral intentions as much as those high in the DG trait. These results reinforce the findings of Study 1.

However, both Studies 1 and 2 used self-report questionnaires and correlation analysis. The next study was designed to find experimental support for the hypothesis that social support had a compensatory function for individuals low in the DG trait. We used a new sample and a new DG behavior measure to further test our hypothesis.

## Study 3

In Study 3, we manipulated participants' levels of perceived social support before presenting them a scenario involving behavioral intentions of DG regarding job choice and salary. It was expected that, under low social support conditions, individuals high in the DG trait would be more willing to defer gratification than those low in the DG trait, while under high social support conditions, individuals low in the DG trait would exhibit as much DG behavior as those high in the DG trait.

### Methods

#### Participants

Sixty-five undergraduate students in an advanced chemistry course at a large public university in China were recruited to participate. Among them, 62 (50% male) provided completed data and 3 did not finish the social support manipulation task. The mean age was 21.7 years (*SD* = 0.72 years, range from 20 to 23 years).

#### Materials and procedures

First we measured the DG trait using Ray and Najman's (1986) 12-item GDGQ (α = 0.69). We then primed perceived social support through an experimental manipulation using a method similar to Galinsky et al. (2003) manipulation of perceived power. Half the participants were randomly placed into the high social support condition and were instructed to “write in detail a difficult situation in which your family or friends accompanied you, and how it made you feel.” Participants were also asked to write the names of five people whom they could rely on. In contrast, participants in the low social support condition were instructed to “write in detail a difficult situation which you had to face all by yourself and how it made you feel.” After completing their responses, participants in both conditions responded to three items for a manipulation check: “Right now, I am feeling that my family/friends really try to help me,” “Right now, I am feeling I can depend on my family/friend when things go wrong,” and “Right now, I am feeling there is a special person in my life who cares about my feelings” (1 = *strongly disagree*, 7 = *strongly agree*). We combined responses to the items to form a single index of perceived social support (α = 0.65). As intended, participants in the high social support condition (*M* = 5.98, *SD* = 1.06) reported more social support than those in the low social support condition (*M* = 5.51, *SD* = 0.85), *F*_(1, 60)_ = 2.85, *p* = 0.06.

Subsequently, participants completed a form describing the following scenario that was modified from Kuhlen and Monge (1968): “A friend of yours of your own age has had two jobs offered to him/her. The two offers were: A) a job with higher immediate salary and B) a job starting with lower salary but with the possibility of much higher income in the future.” Participants then responded to the question “How certain are you that this is what you would advise?” on a 7-point Likert scale (1 = *very certain to advise A, 4* = *not certain, 7* = *very certain to advise B*). This job choice, as suggested by Twenge et al. (2003), can serve as a measure of DG behavioral intention, where choice A favors a short-term gain at the expense of long-term gain, whereas choice B favors long-term gain over short-term gain. Choice B was thus scored as a higher DG behavioral intention. After completing the experiment, participants were thoroughly debriefed and thanked for their participation.

### Results and discussion

**Figure 3:**
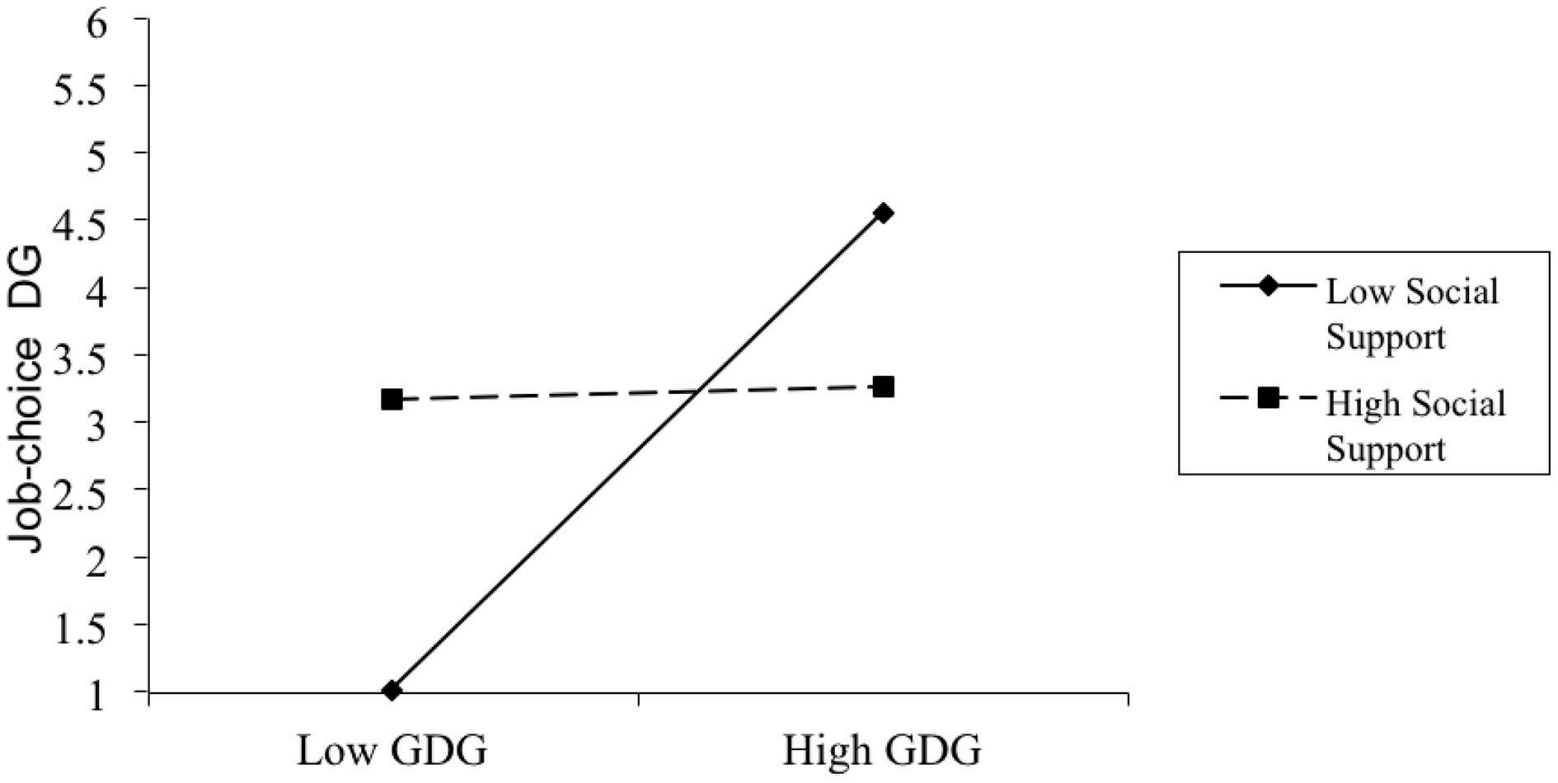
**Interaction between GDG and social support manipulation on job-choice DG**. Note: low = 1 SD below the mean; high = 1 SD above the mean.

Table 1 displays the means, standard deviations, and correlations for all variables in Study 3. In this study, DG trait was positively related to job-choice DG behavioral intention. We conducted a hierarchical multiple regression analysis to test our hypothesis. The data from the GDGQ were centered for a moderation test, and the social support manipulation was coded as 0 for the low social support condition and 1 for the high social support condition. The regression analysis consisted of 3 steps. In Step 1, job-choice DG was regressed on the control variables including gender and age. The results from this step failed to reach significance (*R*^2^=0.07). In Step 2, job-choice DG was regressed on social support and GDG. The results of this step were statistically significant, *R*^2^ = 0.18; ▵*R*^2^ = 0.12, *F*_(4, 56)_ = 3.16, *p* < 0.05. Results showed that GDG could significantly predict DG behavioral intention such that as GDG increased, job-choice DG also increased (β = 0.57, *p* < 0.01). In Step 3, job-choice DG was regressed on the two-way interaction between GDG and social support. The interaction term significantly predicted job-choice DG, *R*^2^ = 0.25; ▵*R*^2^ = 0.06*, F*_(5, 54)_ = 3.62, *p* < 0.05 (see Figure 3). Consistent with our hypothesis, the simple slopes analysis indicated that GDG had a strong impact on job-choice DG for the low social support group (β = 1.77, *p* < 0.01), but did not have a significant impact on job-choice DG for the high social support group (β = 0.05, *p* = 0.09).

In sum, the results of Study 3 supported the hypothesis that social support moderated the relationship between the DG trait and behavior. When there was a high level of perceived social support from friends or family, participants who were low in the DG trait displayed job-choice DG behavioral intentions that were very similar to those who were high in the DG trait. However, when low-DG trait participants did not perceive the social support, they displayed less job-choice DG behavioral intentions than high DG-trait participants. These results confirmed the findings of Studies 1 and 2, suggesting that social support can be a moderator in the DG trait-behavior relationship, such that the people low in the DG trait can show more DG behavioral intention when perceived social support, even if recalled from memory, is made salient.

## Study 4

In the previous study, we investigated how the relationship between DG trait and DG behavior was affected by experimentally manipulated social support. In Study 4, we clarify some confused variables and confirm the function of social support. First, according to previous research, self-image may influence cognition and help people to attain positive outcomes (Steele et al., 1993; Amy and William, 2006). Therefore, we control for self-esteem to rule out the explanation that primed social support could allow people to maintain a self-image that is discrepant with their actual behaviors. Second, previous research has demonstrated that negative moods prompt people to prefer short-term rewards as a mood-regulation strategy (Tice et al., 2001; Fujita et al., 2006). Therefore, we control PANAS to rule out the possibility that the manipulation of perceived social support could be re-interpreted as a manipulation of mood. Finally, the biggest difference between Studies 3 and 4 is that the dependent variable in Study 4 is a choice scenario in which participants make a decision for themselves instead of providing advice for others. Our hypothesis is that under low social support conditions, individuals high in the DG trait would be more willing to ask others to defer gratification than those low in the DG trait, while under high social support conditions, individuals low in the DG trait would advice others to prefer job-choice DG as much as those high in the DG trait.

### Methods

#### Participants

We recruited 64 undergraduate students (47% male) who were attending classes at a large public university in China. Their mean age was 21.0 years (*SD* = 0.89 years, range from 20 to 23 years).

#### Materials and procedures

We first measured the DG trait using Ray and Najman's (1986) 12-item GDGQ (α = 0.70), and we then applied the same method used in Study 3 to manipulate perceived social support. In the manipulation check (the same as described in Study 3), participants in the high social support condition (*M* = 5.98, *SD* = 0.72) reported more social support than those in the low social support condition (*M* = 5.31, *SD* = 1.30), *F*_(1, 62)_ = 8.31, *p* = 0.01 (α = 0.78).

Next, participants completed a DG choice directly involving money which is widely used to measure DG behavioral intension (Funder and Block, 1989; Wulfert et al., 2002). The question goes like this:” If you give up getting ¥10 at once in order to obtain ¥50 in the future, how long are you willing to wait at most: (1) 1 day, (2) 1 week, (3) 1 month, (4) 2 months, (5) 3 months, (6) 6 months, (7) 12 months.” Participants then rated themselves using Watson et al. (1988) 20-item Positive and Negative Affect Scale (PANAS) on a five-point scale (PA α = 0.60; NA α = 0.75), and Rosenberg (1965) 10-item Self-esteem Scale on a 7-point scale (e.g., “I feel that I'm a person of worth”; α = 0.69). After completing the experiment, participants were thoroughly debriefed and thanked for their participation.

### Results and discussion

**Figure 4:**
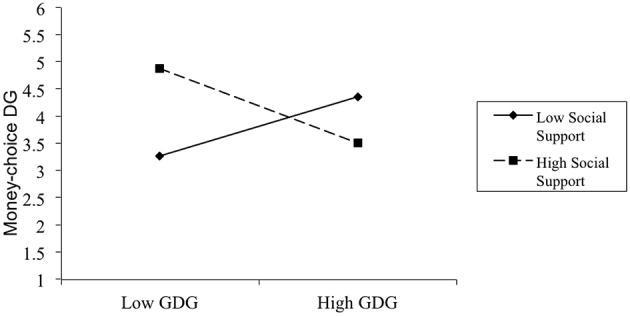
**Interaction between GDG and social support manipulation on money-choice DG**. Note: low = 1 SD below the mean; high = 1 SD above the mean.

Table 1 displays the means, standard deviations, and correlations for all variables in Study 4. In this study, DG behavioral intention was positively correlated with age, but was not related to DG trait, affect, or self-esteem. The data from the GDGQ, PANAS and Self-esteem Scale were centered for a moderation test, and the social support manipulation was coded as 0 for the low social support condition and 1 for the high social support condition. We found that participants did not report statistically different negative affect, positive affect, or self-esteem between the high and low social support conditions. Next, we conducted a hierarchical multiple regression analysis to test our hypothesis. In Step 1, money choice DG was regressed on the control variables including gender, age, positive affect, negative affect, and self-image. The results from this step failed to reach significance, and these control variables accounted for 9.0% of the variance in money-choice DG; age could significantly predict DG behavioral intention such that the older participants would wait longer than the younger ones. In Step 2, money-choice DG was regressed on social support and GDG. The results of this step again were not statistically significant, ▵*R*^2^ = 0.04. Results showed that GDG could significantly predict DG behavioral intention such that as GDG increased, money-choice DG also increased (β = 0.57, *p* < 0.01). In Step 3, money-choice DG was regressed on the two-way interaction between GDG and social support. The interaction term significantly predicted money-choice DG, *R*^2^ = 0.19; ▵ *R*^2^ = 0.06*, F*_(5, 58)_ = 1.64, *p* < 0.05 (see Figure 4). The simple slopes analysis indicated that under the low social support condition, high trait participants had significantly more money-choice DG behavioral intentions than low trait ones (β = −0.69, *p* < 0.05), but for the high social support group, GDG did not have significant impact on money-choice DG (β = 0.38, *p* > 0.10).

To summarize, the results of Study 4 once again confirmed that social support affected low DG trait individuals to show DG behavioral intentions, even when mood and self-esteem were controlled. However, the difference in DG behavioral intention between high and low DG trait participants under the high social support condition was not the same as in the three studies presented above In Study 4, manipulation (*1* = *high social support, 0* = *low social support*) was negatively correlated with DG trait which means that participants in the high social support group had lower DG trait than the other group. Therefore, the lower trait participants who received high social support showed dramatically higher DG behavioral intention than the higher trait ones.

## Study 5

Study 5 took place in a laboratory and included a new measure of the dependent variable. Unlike Studies 1–4 that used domain-specific self-report measures, we assessed participants' actual DG behavior by observing whether participants actually waited for 30 min to get a bigger reward. We expected that DG trait and perceived social support would interact in predicting whether participants engaged in a time-consuming and boring task for more money. Specifically, we anticipated that individuals high in the DG trait would be more likely to engage in a DG task than those low in the DG trait when we have them do a simple task (control group), whereas individuals low in the DG trait would exhibit DG behavior similar to that of high DG trait individuals when we have them to write a social support story (social support group).

### Methods

#### Participants

Thirty-two students (38% male) at Peking University were recruited from the campus Bulletin Board System Their mean age was 22.1 years (*SD* = 2.81, range from 18 to 28 years). In return for their participation, students received ¥5–15 depending on their task performance.

#### Procedure

We recruited participants by posting an advertisement on the campus BBS announcing that students could receive ¥5 after completing a 10-min paper and pencil test. We did not mention that there was another experiment with an additional reward after the 10-min test.

##### Independent variable

When participants came to the laboratory, we asked them to complete the GDG scale (Ray and Najman, 1986; α = 0.91). In order to avoid making them aware of our purpose and feel the test was too simple, they were asked to do a number-eliminating task (for example, crossing off as many numbers containing the digit 3 as possible in a random number table in 3 min. We also had participants rate their income or pocket money per month on a 7-point Likert scale (1 = *extremely little*, 7 = *extremely much*).

##### Moderating variable

Participants then were randomly assigned to the social support group or control group. In the social support group, we asked them to write about an experience of being supported by family or friends to overcome difficulties. In the control group, they were old to write about some uses of new energy sources. These tasks took 10 min.

##### Dependent variable

After completing these tasks, we told the participants that they had finished the experiment and could get ¥5, but that there was another experiment recruiting participants in which they also could participate. This experiment would measure heartbeat and pulse, and they would have to sit still on a chair for 30 min in order to get precise data. It was noted that they could not move, use a phone, sleep, or read If they completed the task, that is, meeting all the requirements and not quitting before the end, they would get¥15 in total. However, they could choose not to participate in the second study and leave with the ¥5. Regardless of whether they chose to stay or not, we measured their emotional mood by using PANAS (Watson et al., 1988; PA: α = 0.90, NA: α = 0.84) and did a manipulation check for perceived social support using two items (“Right now, I am feeling that my family/friends really try to help me” and “Right now, I am feeling I can depend on my family/friend when things go wrong”; 1 = *strongly disagree*, 7 = *strongly agree;* α = 0.95) before they left the laboratory.

Those participants who decided to stay and fulfilled the requirement of sitting still received the bigger payment. We did not actually measure heartbeat or pulse, and we explained our real purpose, that is, we wanted to investigate whether they would stay for a period of time in order to get a greater reward.

### Results and discussion

Table 1 displays the means, standard deviations, and correlations for the variables that were assessed in Study 5. GDG was positively related to PA (*r* = 0.65, *p* < 0.01) and negatively related to NA (*r* = −0.36, *p* < 0.05). However, GDG trait was not related to the DG task. In the manipulation check, participants in the social support group (*M* = 6.28, *SD* = 0.59) reported more social support than those in control group (*M* = 5.51, *SD* = 1.30), *F*_(1, 30)_ = 6.37, *p* = 0.04.

Because the dependent variable is dichotomous (1 = *participating in the delay task*. vs. 0 = *not participating in the delay task*), we used logistic regression analyses to test our hypothesis. Gender, age, income, GDG, PA, and NA scores were centered and entered in step 1. The first step in this analysis was not significant. The interaction term between DG trait and social support condition was entered in Step 2. This step was significant, χ^2^ = (7, *N* = 32) = 25.55, *p* < 0.01. ▵Nagelkerke *R*^2^ = 0.40. Logistic regression revealed a significant interaction (Wald's test of significance = _6.10, *p* = 0.01). Chi-square analyses conducted within the control condition revealed a significant main effect of DG trait on DG behavioral task, χ^2^ = (6, *N* = 16) = 22.18, *p* < 0. 01, but not within the social support condition, χ^2^ = (6, *N* = 16) = 11.90, *p* > 0.05. These results support our hypothesis: when participants wrote a neutral essay (about energy), high DG trait individuals were more likely to engage in an additional boring and time-consuming task for more money than were low DG trait individuals. However, when participants were asked to write about social support in their lives, they were more likely to engage in the DG task regardless their level of the DG trait.

Taken together, these results are in agreement with those of Studies 1 through 4 and support our hypothesis that social support can help people low in the DG trait to show relatively high DG behavior. Furthermore, in Study 5 we replaced the self-report measure of DG behavioral intention with actual DG behavior, thus providing more solid evidence of the effect.

## General discussion

Despite advances in understanding the effects of DG on human development, little research has studied particular conditions under which individuals low in DG nonetheless can act similarly to those high in DG. In our five studies incorporating 698 participants, we provided strong evidence that under high social support conditions, individuals who are low in the trait display DG behavioral intention and actual DG behavior (Study 5) at least at the same level as those who are high in the DG trait. We found that social support can moderate the relationship between the DG trait and DG behaviors in many domains, including vocational, academic, job-choice, and monetary-choice. Further, this result occurred both when social support was only measured and when it was manipulated, and the finding occurred in both naturalistic and laboratory settings.

By using measures of both the DG trait and DG behavioral intentions or behavior, our studies contribute to the current understanding of DG. Our findings particularly challenge the importance of the DG trait. Existing literature shows that DG as a personality trait is shaped in early childhood and can predict adult achievement (Mischel et al., 1988; Shoda et al., 1990; Duckworth and Seligman, 2005). But this outlook may be harmful for those who are low in the DG trait, since it would mean that they are less likely to be successful throughout life. In addition, currently there may be too much emphasis on early education and the fostering of this trait during childhood, particularly if such emphasis causes people to ignore the influence on adult DG behavior of post-childhood experiences and the environment.

However, our research found that the DG trait-behavior relationship in adolescents did not reappear among adults, suggested by the low correlations between GDG and specific-domain DG behavior in most of our studies (Studies 1, 4, and 5). The research context in Study 2 was behavior in schools, which is similar to previous research (Mischel et al., 1992; Duckworth and Seligman, 2005). In academic domains, performance depends mainly on individual aspects of the individual (e.g., hard working, which is close related to DG). But in the workplace (VDG) and social environment (Money-choice DG), people's performance will be affected by many factors (e.g., chance or opportunity), not only DG. In support of the importance of contextual influences, our findings demonstrate that one factor producing DG behavior in adults is the presence of social support, with which even those low in the DG trait, exhibit DG behavior consistent with that of adults high in the trait.

Our findings therefore suggest that having a low level of the DG trait as an adult does not necessarily lead to less success. For example, in Study 1, the DG trait was not a significant predictor of VDG (β = 0.03, *p* > 0.10; see Table 2). We documented that people with low DG trait can still show VDG behavioral as much as those with high DG trait when social support is available, they may gain equal career success. Since VDG, involves certain behaviors that reflect diligence and responsibility (e.g., “It is worth working for a relatively longer period of time for a higher position”), people who display VDG may be given more opportunities for promotion and furthering their careers, thus leading to an overall more successful work life. Recalling our argument that DG may be considered a form of self-regulation, our result is consistent with previous studies which found that some conditions could counteract the loss of self-control resources, including rest (Baumeister et al., 2000), autonomy (Moller et al., 2006), and visualizing an energizing significant other (Knowles and Finkel, 2005).

Our research also helps to explain a finding in a previous study. It has been commonly assumed that the older a person is, the more cautious and long-term oriented he or she will be. However, an empirical study showed that older participants were actually more reluctant to delay gratification in their careers (Pogson et al., 2003). Similarly, our first study found that age was positively related to the DG trait, but negatively related to VDG, and that the trait was not significantly related to VDG behavioral intention (see Table 1). Our finding contributes to the explanation of the incongruence between the assumption that older people are more inclined to delay gratification and Pogson et al.'s finding. It may be true that people become higher in a general DG trait as they age, but they may also come to seek certain types of gratification without delay since their time is limited. In addition, in Study 1 we also found that the higher the educational level and position and the longer the length of service employees had, the higher was their DG but not their VDG. These findings reinforce the importance of considering DG in specific contexts and in specific domains.

The present research also advances methodology in measuring adults' DG. In previous studies, it was measured solely through questionnaires, whereas in Study 5 we modified the paradigm used with children, providing our adult participants the choice between an immediate ?5 reward and a later ¥15 reward, and we designed a frustrating and difficult waiting period, as in the children's paradigm. In this way, we replaced the typically-used self-report index with a behavioral indicator. This approach may contribute to future researchers' adopting more experiential methods in the study of adult DG.

Also importantly, the present research sheds light on the mechanism of the occurrence of DG behavior. All five studies demonstrated that people low in the DG trait can show DG behavioral intentions—or in Study 5, behavior—when they have the proper social support, meaning that social support can be a substitute energizer for those who are low in the DG trait, providing a powerful element to produce DG behavioral intention. Our results also can be explained by the recent finding that social support is a unique trigger of additional effort and performance (Wessolowski et al., 2014). Although externally imposed control can substitute for self-control in pursuing long-term goals (Fishbach and Trope, 2005), the mechanism of social support is different from external control. In Fishbach's study, participants' behaviors changed when they were made? Aware of others' supervising or paying them for their behavior. We instead counter-balanced the order of our measures: in Studies 1 and 2, participants first completed the DG behavioral intention questionnaires and then the perceived social support questionnaire, whereas the opposite order was used in Studies 3, 4, and 5. Thus, we found that social support can help people low in the DG trait to show high DG behaviors regardless of whether or not their attention is drawn to the social support. Furthermore, we ruled out other possible moderators such as moods and self-image. In our last study, perceiving a lack of social support did not induce negative affect, which has previously been shown to be related to DG behaviors (Tice et al., 2001). Neither did social support promote a change in self-image which may influence participants' cognitions and help them to display positive outcomes (Amy and William, 2006).

Our research has several limitations. First, although we conducted a behavioral experiment in Study 5, the first four studies also relied on self-report measures, and the GDGS and the domain-specific scales were used at the same time. Both more prospective studies and a greater variety of indexes would strengthen the validity and generalizability of our findings. For example, collecting data from different source, such as supervisor ratings of vocational delay of gratification, could help avoid possible common method variance, as would incorporate more actual behavior as data. Regarding social support, we used the overall MSPSS rather than its three subscales; further studies may differentiate among types of social support (from families, friends and important others), as the source of social support may be relevant, particularly in different domains. Despite these limitations, our studies went beyond previous research by studying DG behaviors in adults in the context of both the DG trait and an important environmental factor, and it revealed the effects of social support in moderating the relationship between the DG trait and behavioral intention. Our studies enrich the current understanding of DG by showing a boundary condition of the DG trait-behavior relationship. Practically, our finding may be useful for educational systems, families, and a wide range of social programs that may help adolescents and low in the DG trait to display DG behaviors that are, in turn, beneficial for personal development and success. The findings are also instructive for organizational management in that social support may be used to trigger employee DG behaviors, particularly when organizations are unable to provide close supervision or external rewards such as better salaries or promotions. In addition, our research shows that the popular emphasis on childhood DG's predicting adult success should not limit the attention paid to shaping DG behavior in adulthood.

## Author contributions

XL is the maim paper design and paper maker. LW is Liu's supervisor and guided the whole experiments. JL revised the Language and format for paper.

## Funding

National Natural Science Foundation of China (Grant Nos. 71202021, 71402035).

### Conflict of interest statement

The authors declare that the research was conducted in the absence of any commercial or financial relationships that could be construed as a potential conflict of interest.
